# Root system architecture analysis in *Mesembryanthemum crystallinum* (ice plant) seedlings reveals characteristic root halotropic response

**DOI:** 10.1242/bio.052142

**Published:** 2021-03-29

**Authors:** Mayuko Otsuka, Hikaru Kato, Shyota Yamada, Tatsuhiko Nakayama, Satomi Sakaoka, Atsushi Morikami, Hironaka Tsukagoshi

**Affiliations:** Faculty of Agriculture, Meijo University, 1-501 Shiogamaguchi, Tempaku-ku, Nagoya, Aichi 464-8601, Japan

**Keywords:** *Mesembryanthemum crystallinum*, Halotropism, Root system architecture, Auxin

## Abstract

One of the major environmental stress factors that affect root growth is salinity. *Arabidopsis thaliana*, a glycophyte, shows halotropism, whereby it alters the direction of root growth in a non-gravitropic pattern to evade high soil salinity. Asymmetric auxin distribution regulated by the relocation of auxin-efflux carrier proteins is a key cellular event in the halotropic response. However, there are no reports of halotropism in halophytes. Here, we investigated root growth traits in *Mesembryanthemum crystallinum* (ice plant), under high salinity conditions. We hypothesized that ice plant roots would show halotropic responses different from those of *Arabidopsis*. Notably, similar to halotropism observed in *Arabidopsis*, ice plant roots showed continuous root bending under salinity stress. However, the root elongation rate did not change in ice plants. Expression analyses of several genes revealed that auxin transport might be partially involved in ice plant halotropism. This study enhances our understanding of halophyte root adaptation to high salinity stress.

## Abstract (precis)

**Summary:** Halotropism is one of the key root growth traits that facilitates adaptation to saline soil conditions. The halophyte *Mesembryanthemum crystallinum* (ice plant), exhibited halotropic root growth under high salinity conditions.

## INTRODUCTION

In plants, the roots constitute the supportive structure for their aerial plant body. Additionally, they absorb nutrients and water from the soil, and facilitate responses to changes in the surrounding environment. The roots generally grow in the direction of the gravitational pull, a tropic response called gravitropism that facilitates spreading of the root system in the soil and the establishment of a wide root network. However, environmental stress factors may impair root development.

One of the major abiotic stress factors that affect crop production is salinity. Generally, severe salinity stress inhibits root growth, even to the point of plant withering. In addition, salinity stress inhibits water uptake by increasing osmotic stress and inhibiting enzyme activities (Blumwald, 2000; Munns and Tester, 2008). Even under moderate salinity stress, plants display alterations in the root system architecture (RSA) to facilitate optimal root activities (Julkowska et al., 2014). For example, in *Arabidopsis thaliana*, root growth does not follow gravity when the plant is grown in a high-salinity medium (Sun et al., 2008). In glycophytes, the root gravitropic response changes via alteration in the RSA under high salt conditions. Thus, for example, tomato and sorghum roots grow away from saline soil, against gravity (Galvan-Ampudia et al., 2013). This tropic response, called ‘halotropism’, allows plants to minimize their exposure to high-salinity conditions. Therefore, halotropism is a key trait that facilitates plant adaptation to saline conditions via the regulation of root development. When roots perceive high salt levels, PIN2, one of the auxin transporters that determine auxin distribution, relocates to the side of the root exposed to salt stress. This relocation causes an asymmetric auxin flow under salt stress (Galvan-Ampudia et al., 2013). PLDζ2 plays an important role in PIN2 relocation during the endocytosis of the membrane proteins (Li and Xue, 2007); furthermore, PLDζ1 participates in PIN2 relocation under salt stress as a part of the halotropic response (Korver et al., 2020). Besides PIN2 relocation, the localization of AUX1, which is active in the import of auxin into cells, is also critical for the occurrence of root halotropic response (Korver et al., 2020). The findings indicate that the alteration in auxin polarity is essential for root halotropic activity. In addition, a genome-wide association study using 333 *Arabidopsis* accessions identified several genetic factors associated with root halotropism (Deolu-Ajayi et al., 2019). Furthermore, three genes were characterized, namely, *WRKY25*, *CHX13*, and *DOB1*, which were not involved in auxin distribution (Deolu-Ajayi et al., 2019). The study revealed that other factors are a part of the regulatory mechanism of halotropism, besides auxin distribution.

Halophytes, such as ice plant (*Mesembryanthemum crystallinum*), show salinity tolerance. The ice plant continues to grow in saline soils with salt concentrations exceeding 450 mM NaCl, which is higher than the salinity level in seawater (Adams et al., 1998). Furthermore, the roots of ice plant seedlings can grow in soil with salinity levels of >140 mM NaCl that almost halt the root growth in *Arabidopsis* (Tsukagoshi et al., 2015). In addition, we constructed an mRNA expression database for ice plant roots that were treated with NaCl at several concentrations to identify salt stress-responsive genes (Tsukagoshi et al., 2015). Using this database, we identified a Na^+^ transporter, McHKT2, which was involved in salt stress tolerance (Nishijima et al., 2017). However, we did not find an auxin-related gene ontology (GO) category among the enriched GOs in our database. These findings indicated that ice plant roots may not alter auxin signals under high-salt conditions. In addition, we did not observe non-gravitropic root development in a high-salt medium (Tsukagoshi et al., 2015). Overall, these findings warrant further investigation of halotropism in ice plant roots to enhance our understanding of salt tolerance in halophytes.

In this study, we conducted an RSA analysis in ice plants grown under high-salinity conditions. The results revealed that ice plant roots showed halotropic root growth in a gel with a high salt concentration, but not on the surface of a solid medium.

## RESULTS

### Root bending pattern of ice plants grown in a high NaCl-containing medium

**Fig. 1.**
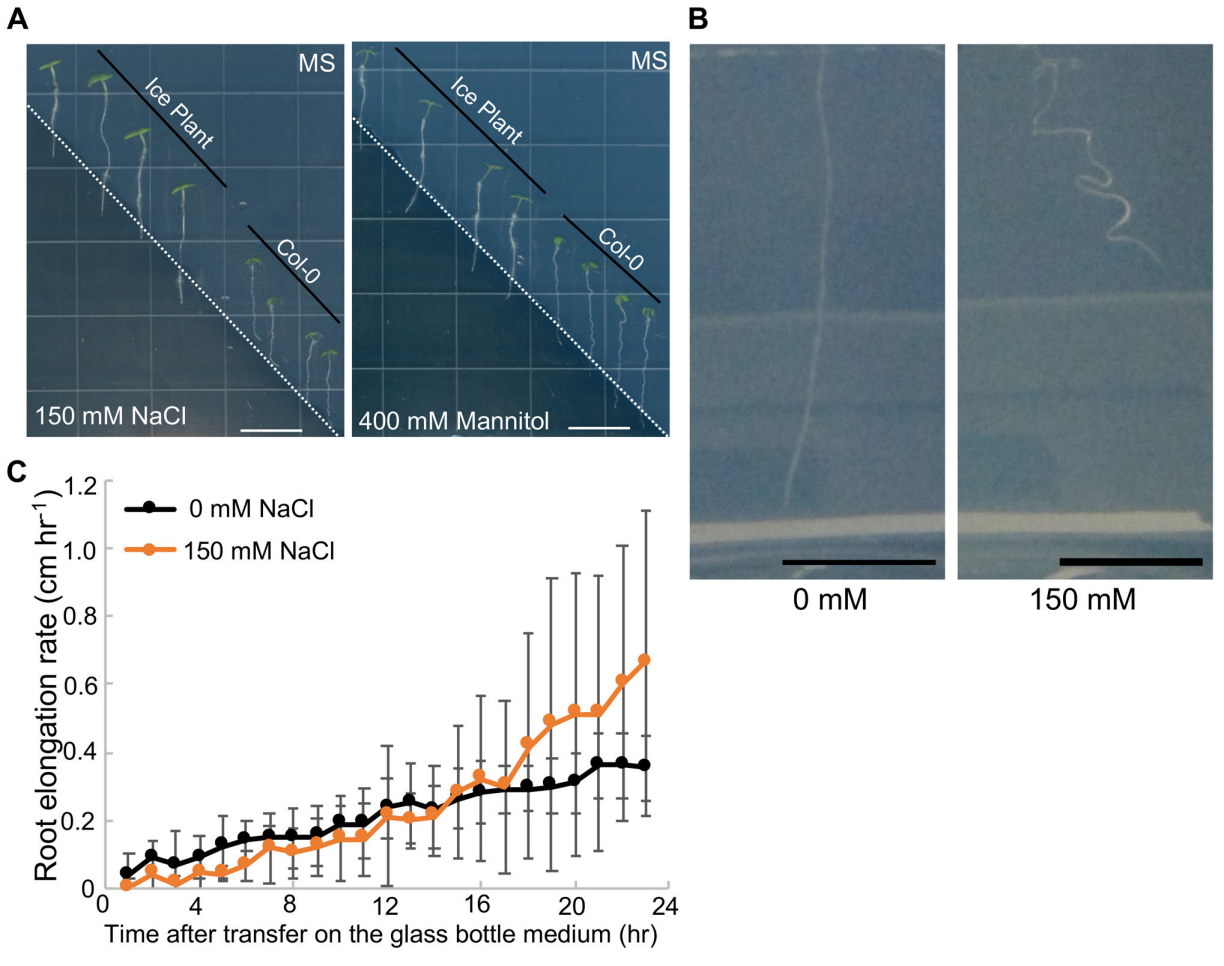
**Difference in ice plant root growth characteristics between treatments without NaCl and with high NaCl.** (A) Ice plant and *Arabidopsis* root growth in the halotropism assay plates, which contained either no NaCl or 150 mM NaCl (left panel) and no NaCl or 400 mM mannitol-containing medium (right panel). White dashed line: the border between the MS and the 150 mM NaCl or 400 mM mannitol medium. Scale bars: 1 cm. (B) Ice plant root grown in solid medium containing 0 and 150 mM NaCl for 3 days after transfer of ice plant seedlings onto solid medium in a glass bottle. Scale bars: 1 cm. (C) Root elongation rate in ice plants grown in solid medium containing 0 and 150 mM NaCl was measured every 1 h for 24 h. Data are presented as mean±s.d. (*n*=5). Black line, 0 mM NaCl medium; orange line, 150 mM NaCl-containing medium.

We analyzed the root growth patterns of ice plant using a split gel system, which is called the halotropism assay plate, on a solid gel without NaCl on one side and with 150 mM NaCl on the other (Galvan-Ampudia et al., 2013). *Arabidopsis* Col-0 ecotype was transferred to the same halotropism assay plate as the halotropism control. *Arabidopsis* roots showed a high degree of halotropism; however, the roots of ice plants grown in the part with salt-containing solid gel grew straight (Fig. 1A). Overall, ice plant seedlings did not show halotropic growth on the halotropism assay plates. Furthermore, osmotic stress induced by 400 mM mannitol affected neither ice plant nor *Arabidopsis* root growth (Fig. 1A).

**Fig. 2.**
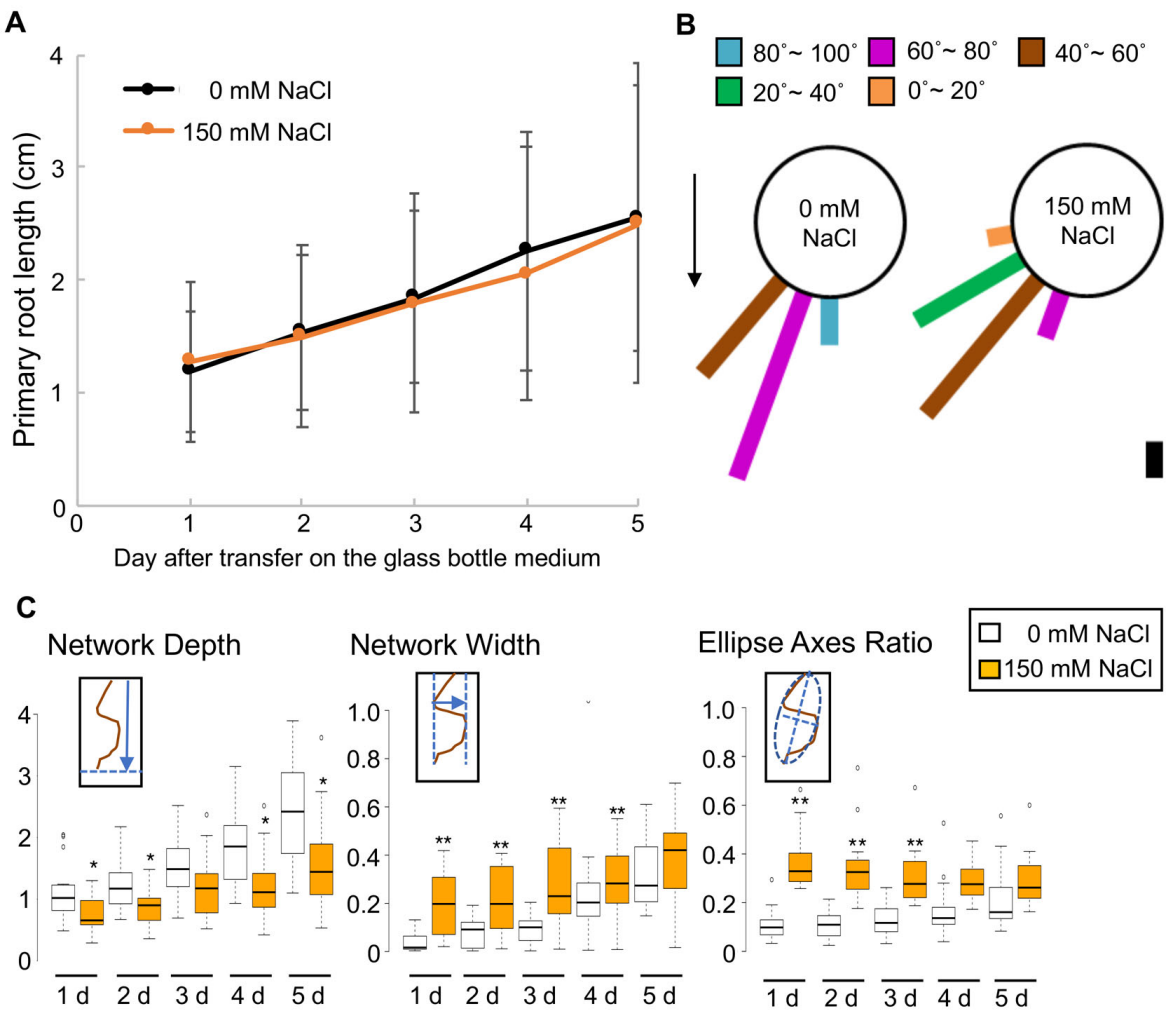
**RSA analysis of the roots of ice plants grown in NaCl-containing solid medium.** (A) Root length of ice plants grown in 0 and 150 mM NaCl-containing solid media was measured every day for 5 days after transfer of the ice plant seedlings onto solid medium in glass bottles. Data are presented as means±s.d. (*n*=15). Black line, 0 mM NaCl medium; orange line, 150 mM NaCl-containing medium. (B) Growth direction of roots 3 days after transfer of ice plant seedlings onto solid medium in glass bottles containing 0 and 150 mM NaCl, at intervals of 20° (*n*=15). The arrow indicates the direction of gravity. Scale bar: 10% roots. (C) ‘Network Depth’, ‘Network Width’ and ‘Ellipse Axes Ratio’ RSA traits calculated using GiA-roots. Each RSA trait was calculated every day for 5 days after transfer of ice plant seedlings onto solid medium in glass bottles. **P<0.01 and *P<0.05, determined using Student's *t*-test compared with the 0 mM treatment everyday (*n*=15). White boxes, 0 mM NaCl medium; orange boxes, 150 mM NaCl-containing medium. Schematic drawings in small boxes in each graph indicate the measurement of each RSA. Brown curves, plant roots; blue arrows, measurement of ‘Network Depth’ and ‘Network Width’; blue dotted lines in ‘Ellipse Axes Ratio’, the minor and the major axes of the best fitting ellipse.

To investigate how halophyte roots grow under high NaCl conditions, we observed ice plant root growth in a medium contained in glass bottles (Fig. S1). Ice plants were germinated on horizontal Murashige and Skoog (MS) plates for 3 days; then, the germinated plants were transferred onto MS medium in glass bottles containing 0 mM or 150 mM NaCl. Three days after transfer, we obtained time-lapse images of seedlings in each glass bottle at 1-h intervals for 72 h (Fig. 1B; Movies 1 and 2). Notably, the roots of seedlings in NaCl-containing medium did not grow straight; instead they continuously bent in the medium. As we hypothesized that NaCl would inhibit root growth, we measured root elongation under all treatment at 1-h intervals for 24 h (Fig. 2C). However, the root elongation rates under the 150 mM NaCl treatment tended to be higher than that under the control conditions, and there were no considerable differences between the 0 mM and the 150 mM NaCl treatments. Overall, ice plants exhibited halotropism when the plants were grown in NaCl-containing medium, but NaCl did not inhibit root elongation.

### RSA analysis of ice plants grown in NaCl-containing medium

To quantify root growth in the ice plants, we conducted the RSA analysis of the roots of ice plants grown in a medium containing 150 mM NaCl. Photographs were taken every day for 5 days, starting from day 3 after transferring the 3-day-old plants that germinated on vertical plates. Initially, we measured root length and root growth angle. There were no major differences in the root length between the control and NaCl-treated plants (Fig. 2A). However, the root growth angle of plants grown in NaCl-containing medium was higher than that of plants grown in the control medium (Fig. 2B). Overall, the roots of ice plants in high-salt medium showed impaired gravitropism, which is rather equivalent to a halotropic response. Notably, the root growth traits of the seedlings grown in a high-salt medium were different from those of seedlings grown on the surface of the vertical plates.

### Quantification of RSA of root halotropism in ice plant

Besides the root length and root angle, we quantified root traits using GiA-roots (Galkovskyi et al., 2012). GiA-roots can calculate 2-D RSA features of individual roots. Among 19 features that GiA-roots supported, we used three features, namely, ‘Network Depth’, ‘Network Width’, and ‘Ellipse Axes Ratio’. ‘Network Depth’ calculates RSA depth from the top to the lowest network or indicates the root growth area downward into the soil profile. ‘Network Width’ calculates RSA width from the left-most network to the right-most network in a horizontal direction; that is, it indicates the spread of root system. In turn, ‘Ellipse Axes Ratio’ calculates RSA growth angles based on the ratio of the minor axis to the major axis of the best-fitting ellipse of the RSA. As for ‘Network Depth’, the RSA of the NaCl-treated seedlings showed significantly lower depth from day 1 of measurement (Fig. 2C). The results indicated that ice plant roots did not grow deep in the NaCl-containing medium. However, ‘Network Width’ showed that the RSA of seedlings grown in the 150 mM NaCl-containing medium was significantly broader than that of seedlings grown in the 0 mM NaCl medium, until day 4 of measurement. However, ‘Network Width’ between the 0 and the 150 mM NaCl treatments at day 5 was comparable (Fig. 2C). As the roots in the 0 mM NaCl medium grew diagonally, ‘Network Width’ presented high values even in the control treatment at later measurement stages (Fig. 2C). With regard to ‘Ellipse Axes Ratio’, the roots of NaCl-treated seedlings presented higher values than the roots of control seedlings (Fig. 2C). The roots of seedlings in the 150 mM NaCl treatment presented the highest ‘Ellipse Axes Ratio’ value in the early stages. Taken together, the roots of plants grown in NaCl medium showed a tightly packed RSA trait. Besides the RSA analysis, the images used for GiA-roots calculations revealed that the roots of plants grown in the NaCl-containing medium showed continuous bending and shallow angles (Fig. S2). The results suggest that halotropism changes the direction of root growth continuously in ice plant, although it does not affect root elongation.

### Exogenous treatment of auxin did not affect halotropism in ice plants

**Fig. 3.**
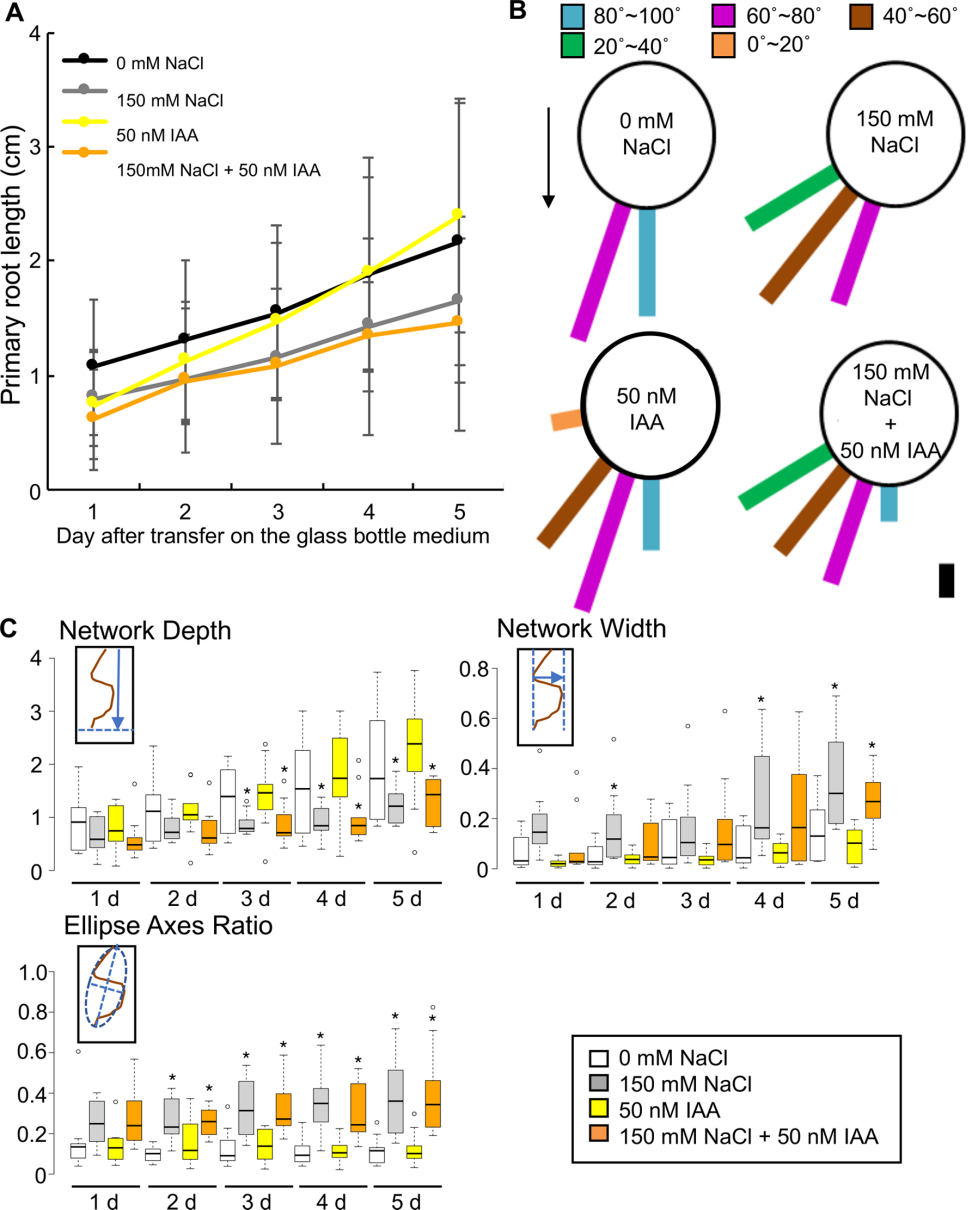
**RSA analysis of the roots of ice plants grown in solid medium added with NaCl and supplemented with 50 nM IAA.** (A) Root length of ice plants grown in solid medium containing 0 mM NaCl (black line), 150 mM NaCl (gray line), 50 nM IAA (yellow line), or 150 mM NaCl supplemented with 50 nM IAA (orange line) was measured every day for 5 days after the transfer of ice plant seedlings onto solid medium in glass bottles. Data are presented as means±s.d. (*n*=10). (B) Growth direction of roots 3 days after transfer of ice plant seedlings onto solid medium, containing 0 mM NaCl, 150 mM NaCl, 50 nM IAA, and 150 mM NaCl supplemented with 50 nM IAA, in glass bottles at intervals of 20° (*n*=10). The arrow indicates the direction of gravity. Scale bar: 10% roots. (C) ‘Network Depth’, ‘Network Width’ and ‘Ellipse Axes Ratio’ RSA traits calculated using GiA-roots. Each RSA was measured every day for 5 days after transfer of ice plant seedlings onto solid medium in glass bottles. **P<0.01 and *P<0.05, determined using Student's *t*-test compared with the 0 mM treatment on each day (*n*=10). White boxes, 0 mM NaCl; gray boxes, 150 mM NaCl; yellow boxes, 50 nM IAA; orange boxes, 150 mM NaCl and 50 nM IAA-containing medium. Schematic drawings in small boxes in each graph indicate the measurement of each RSA. Brown curves, plant roots; blue arrows, measurement of ‘Network Depth’ and ‘Network Width’; blue dotted lines in ‘Ellipse Axes Ratio’, the minor and the major axes of the best fitting ellipse.

We investigated the effect of adding 3-indole acetic acid (IAA) to the medium, in addition to NaCl, on root halotropism in ice plant. The root length of plants in 50 nM IAA-supplemented medium was comparable with that of plants grown in the medium containing 0 or 150 mM NaCl (Fig. 3A). The root growth angle of plants in the 50 nM IAA-medium changed compared to control condition. This indicated that auxin affected the gravitropism of ice plant roots. However, the root growth angle of plants in the 50 nM IAA-supplemented 150 mM NaCl medium did not considerably change when compared with that of plants in the 150 mM NaCl treatment (Fig. 3B). We also conducted an RSA analysis following 50 nM IAA application in the NaCl treatment (Fig. 3C). With regard to ‘Network Depth’, 50 nM IAA did not influence network depth compared with the control conditions, and the addition of 50 nM IAA to the 150 mM NaCl medium did not influence network depth compared with the 150 mM NaCl treatment (Fig. 3C). Although on day 1, ‘Network Width’ of plants grown in 150 mM NaCl medium supplemented with 50 nM IAA was lower than that of plants in 150 mM NaCl medium after day 2. ‘Network Width’ of plants grown in 150 mM NaCl medium and those grown in 150 mM NaCl medium supplemented with 50 nM IAA was comparable (Fig. 3C). The ‘Ellipse Axes Ratios’ value of plants in 50 nM IAA-supplemented medium was comparable with that of plants grown in control conditions, 0 mM NaCl medium, and 150 mM NaCl medium (Fig. 3C). Overall, treatment with a low concentration of auxin did not affect the halotropic activity in ice plants.

### Effect of NaCl on auxin distribution in ice plant roots

We conducted a reverse-transcription quantitative PCR (RT-qPCR) time-course analysis of the induced changes in gene expression. We searched the ice plant mRNA database created following ice plant root RNA-Seq (Tsukagoshi et al., 2015). We used *Arabidopsis* cDNA sequences related to several auxin-related genes as queries and performed BLAST searches in the ice plant database (https://pothos.tsbio.info/Mcr/assembler/blast/language). We selected *PIN*s, which are auxin efflux carrier proteins (Müller et al., 1998; Blilou et al., 2005). Particularly, *PIN3* is considered a key participant in gravitropism in columella cells (Friml et al., 2002; Kleine-Vehn et al., 2010). Moreover, *PIN2* plays a critical role during halotropism in *Arabidopsis* (Galvan-Ampudia et al., 2013). In addition, *ARF19* is one of the transcription factors that mediate responses to auxin in root gravitropism (Okushima et al., 2005; Band et al., 2012), whereas *IAA7* is one of the regulators of functions of the ARF protein (Gray et al., 2001).

**Fig. 4.**
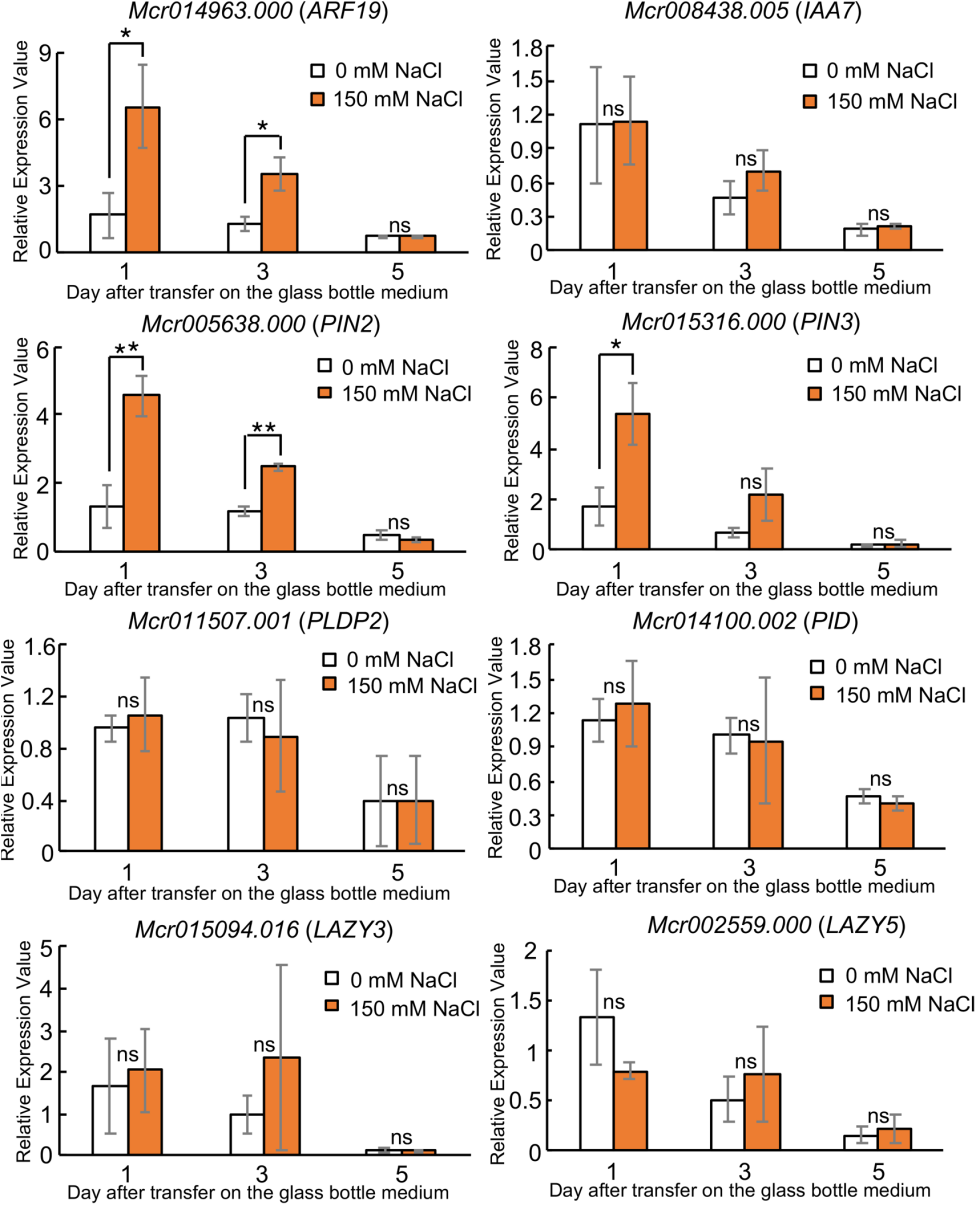
**Expression analysis of auxin-related genes (*ARF19*, *IAA7*, *PIN2*, *PIN3*), *PLDP2*, *PID* and 2 LAZY homologous (*LAZY3* and *LAZY5*) genes.** RT-qPCR analysis of auxin-related genes and *LAZY* homologous genes after 1, 3, and 5 days of the 0 mM and 150 mM NaCl treatments of ice plant roots in solid medium in glass bottles. Data are presented as means±s.d. (*n*=3). **P<0.01 and *P<0.05, determined using Student's *t*-test by comparing the expression levels with the 0 mM NaCl treatment daily. ns; not significant. White boxes, 0 mM NaCl; orange boxes, 150 mM NaCl treatment.

The RT-qPCR results revealed that the *ARF19* homologous gene expression in ice plants was upregulated 1 day after transfer to the glass bottles, and the expression decreased 3 days. At 5 days after transfer, the expression level was comparable with that in the control treatment (Fig. 4). There were no differences in the *IAA7* homologous gene expression level in ice plant roots between the control and NaCl treatments at all time points. However, its expression was higher at 1 day after transfer than at 3 or 5 days after transfer, and this indicated that the auxin levels in the ice plant roots were higher in the initial stages following transfer to the glass bottles. Similar to the *ARF19* homologous gene, the *PIN2* and *PIN3* homologous genes were upregulated 1 day after transfer to the glass bottle medium containing NaCl. This result indicated that NaCl treatment altered *PIN3* expression and that auxin distribution was affected at specific points where the roots began bending. In addition to PIN3, PIN2 would also affect root bending of the ice plant with a change in auxin distribution under high-NaCl conditions.

NaCl treatment induces *PLDζ* (*PLD P2*) and regulates PIN2 trafficking in root tip cells in *Arabidopsis* (Galvan-Ampudia et al., 2013). PLD P2 produces phosphatidic acid (PA) and PA binds to the PID proteins. PA bound by PID regulates PIN2 cellular localization under high-NaCl conditions (Wang et al., 2019). We also investigated *PLD P2* and *PID* homologous gene expression in ice plants. The expression of both genes was comparable between the controls and 150 mM NaCl treatment at all time points. The results indicated that auxin distribution in the ice plant, which is regulated by PIN2 relocation, could be controlled by different mechanisms in *Arabidopsis*.

The *LAZY* genes also regulate root gravitropism (Yoshihara and Spalding, 2017). We investigated *LAZY* homologous gene expression in ice plant roots. As the expression patterns of genes responsible for mediating various auxin responses and those of auxin transport-related genes in ice plants were different from the expression patterns in *Arabidopsis*, we hypothesized that the genes involved in gravitropism might participate in root halotropism in ice plants. We first performed a BLAST search to find *LAZY* homologous genes in our database. We used AtLAZY3 whole amino acid sequence as a query. We found only one cDNA in our database (Tsukagoshi et al., 2015). The LAZY protein family has a 14, highly conserved, amino acid sequence called CLC (Taniguchi et al., 2017). We used the CLC sequence as a query to search for more *LAZY* homologs. We found one *LAZY* homolog candidate cDNA that was the most similar to *AtLAZY5* in addition to the *LAZY3* homolog. The expression of the *LAZY3* and *LAZY5* homolog genes in ice plants between the control and NaCl treatment was comparable. This indicated that NaCl treatment did not influence *LAZY* expression in ice plant, although the ice plant roots displayed impaired gravitropic responses.

## DISCUSSION

We previously reported that ice plant seedlings grown on 140 mM NaCl-containing medium vertically did not exhibit any changes in root growth patterns (Tsukagoshi et al., 2015). Consistent with our previous study finding, here, ice plant roots grew straight even on a halotropism assay plates, with a solid medium without NaCl and with 150 mM NaCl (Galvan-Ampudia et al., 2013). Notably, unlike the root growth patterns on the vertical plates, ice plants grown in the glass bottle showed halotropic root growth traits. As a halophyte, ice plant seemed to evade salinity stress; however, the seedlings maintained growth by altering root growth direction; this suggests that halophytes continuously seek regions with lower salinity stress in the growth environment. The gel system in the glass bottle is more similar to the soil growth conditions than the surface of the solid medium, which indicates that root growth in the soil might have a greater influence on RSA.

An asymmetrical distribution of auxins on the sides of the root, when one side is in contact with the vertical plates, is critical for halotropism in *Arabidopsis* (Galvan-Ampudia et al., 2013). As the roots of ice plants in the medium in the glass bottles can contact NaCl from all directions, asymmetric auxin distribution in the roots is more disturbed in the glass bottles than on the surfaces of vertical plates. Although sole auxin application affected root growth angle, our results indicated that auxin application was not additive of RSA alterations in the 150 mM NaCl treatment. Our results suggest that ice plants may achieve halotropism via molecular mechanisms that are different from the ones in *Arabidopsis*.

However, further expression analysis of auxin-related genes in ice plants grown in the glass bottle systems indicated that auxin might regulate a part of the halotropic response in the ice plant roots. Our gene expression analysis revealed that the *ARF19* homologous gene was induced by NaCl treatment. This explains why the auxin level in the ice plant roots is higher than that under non-saline conditions, that is, *ARF19* expression is induced by auxin (Okushima et al., 2005). The *PIN3* and *PIN2* homologous genes were also induced by NaCl treatment in the ice plant roots. However, *PIN2* expression in *Arabidopsis* reportedly decreased within a few hours under the 150 mM NaCl treatment and that the PIN2 protein level continued to decrease with an increase in the NaCl concentration (Sun et al., 2008). According to our gene expression analyses, PIN2-mediated regulation of auxin distribution in ice plant roots under NaCl treatment was different from that in *Arabidopsis* with respect to halotropism.

The gene expression patterns observed indicate that the RSA results based on ‘Network width’ and ‘Ellipse Axes Ratio’ were similar between the control and NaCl treatments 5 days after transfer to the glass bottle (Fig. 2). The treatment with NaCl could alter auxin distribution in ice plant roots at specific sites where PIN3 is involved in mediating auxin flow, such as in columella cells. Subsequently, such a change would cause bending or disturb gravitropism. In the initial stages after transfer into NaCl- containing media, a high *PIN2* levels would also mediate the alteration in auxin distribution patterns in ice plant roots. However, IAA application to the NaCl-containing media in the glass bottles did not show any considerable effect on continuous root bending. This indicates that high *PIN2* and *PIN3* levels in ice plant roots treated with NaCl could be adequate for the regulation of exogenous auxin. In addition to the alteration in auxin distribution, a high expression of the *ARF19* homologous gene in the initial stages following transfer to the glass bottles might indicate that the auxin level also increases after NaCl treatment. This would be another reason for the lack of an effect of exogenous IAA application in the NaCl-containing media on root growth patterns. Our data suggested that in the initial stages following the transfer of seedling to the glass bottle system, the auxin levels and their distribution in ice plants changed, whereby they showed continuous bending root growth. Future research must test our hypothesis by measuring the auxin levels in ice plant roots. The use of auxin reporter genes, such as *DR5::GFP* in ice plants, might support our hypothesis. However, currently, there is no proper method for ice plant transformation.

We also retrieved *PIN2* and *PIN3* homologous gene expression data from our previous RNA-Seq data set obtained following the growth of ice plant roots on solid medium (Tsukagoshi et al., 2015). In the data set, there were no differences in gene expression levels between the 0 and the 140 mM NaCl treatments. The difference between the current and previous data might be due to the growth conditions of ice plants, including the glass bottle and vertical plate, which again, indicates that the glass bottle conditions for root growth are more similar to the soil conditions.

In this study, ice plant roots exhibited halotropic growth patterns when grown in an NaCl-containing medium. Although the root growth rates did not change in the NaCl medium, the RSA analyses revealed changes in the root growth patterns; this indicated that halophyte roots grow by avoiding high salinity conditions or seek zones with lower NaCl concentrations, where root elongation may continue unimpeded. In contrast to *Arabidopsis*, the application of low concentrations of IAA to an NaCl-containing medium had virtually no effect on the halotropic response of ice plant. The gene expression analysis revealed that putative *PIN2* and *PIN3* homologue genes were upregulated, similar to *ARF*, under high salt concentrations. In addition, ice plant genes that were *Arabidopsis* homologs involved in gravitropism, such as *LAZY*, did not show any changes in expression following treatment with 150 mM NaCl. Our results of gene expression and RSA analyses indicate that even halophytes such as ice plant exhibit root halotropism under high-salinity conditions. In addition, auxins also regulate halotropism in ice plant. The results of this study clearly showed that the mechanism underlying halophyte tolerance to soil salinity involves the salt-induced alteration in root growth patterns.

## MATERIALS AND METHODS

### Plant growth and NaCl treatment

The seeds of *M. crystallinum* and *A. thaliana* Col-0 ecotype were maintained in the dark at 4°C for 1 day, sterilized for 5 min in 25% NaClO and 0.05% Triton X-100, washed three times with sterile water, and sown on 1x MS medium (pH 5.7) containing 1% sucrose and 1% agarose. The seeds were germinated in a vertical orientation in a growth chamber at 22°C under a 16-h light 8-h dark regime.

In the halotropism assay on vertical plates, 5-day-old seedlings were transferred to the halotropism assay plates with 0 mM NaCl medium, and the medium in the bottom right part was replaced by 150 mM NaCl- or 400 mM mannitol-containing medium according to Galvan-Ampudia et al. (2013). We aligned the root tip of each transferred seedling at the border between the two different media. For the glass bottle experiments, 100 ml of MS medium (pH 5.7) containing 1% sucrose and 0.2% Gellan Gum (Wako, Osaka, Japan) with 0 mM NaCl, 150 mM NaCl, 50 nM IAA, and 150 mM NaCl and 50 nM IAA were solidified in 240-ml bio glass bottles (62φ×109 mm; KENIS, Japan). Three days after germination, ice plant seedlings on the vertical MS medium were transferred to the center of the medium in the glass bottles, which were then incubated in a growth chamber at 22°C under a 16-h light 8-h dark regime.

### Imaging of root growth

The supplemental videos were generated using time-lapse images with a digital camera (Visualix Pro2lite; Visualix, Japan) controlled by TCapture (V.4.3.0.602; Tucsen Photonics Co. Ltd, China); the images were captured at 1-h intervals for 72 h. The glass bottles filled with gel were placed in front of the camera. For the RSA analyses, the images of glass bottles with scale bars were captured using a digital camera (PowerShot G12; Canon) everyday, beginning 3 days after the plants were transferred onto the media for 5 days. To avoid reflection from the room light, the glass bottles were placed in a water tank (acrylic plate box, 2 mm thick) according to Iyer-Pascuzzi et al. (2013).

### Root system architecture analysis

Root growth angle into the medium was measured using ImageJ (http://rsbweb.nih.gov/ij). The other RSA traits, including ‘Network Depth’, ‘Network Width’ and ‘Ellipse Axes Ratio’, were calculated using GiA-roots (Galkovskyi et al., 2012). The roots in glass bottle images were traced using pen tools (5 pixel) in Adobe Photoshop (Adobe Systems Inc., San Jose, CA, USA) for submission into GiA-roots (Fig. S1). All output data were exported to MS Excel (Microsoft Corp., Redmond, WA, USA) and used to calculate the RSA traits. Statistical significance was evaluated using a Student's *t*-test.

### RNA extraction and RT-qPCR

Whole roots collected at 1, 3, and 5 days after transfer to medium in the glass bottles were used for RNA extraction using a RNeasy plant mini kit (Qiagen) according to the manufacturer's instructions. The first-strand cDNA was synthesized using ReverTra Ace qPCR RT Master Mix with gDNA Remover (TOYOBO, Osaka, Japan). The RT-qPCR was performed using THUNDERBIRD SYBR qPCR Mix (TOYOBO) on an Illumina ECO qPCR system (Illumina Inc., San Diego, CA, USA). The RT-qPCRs were performed using a reaction mixture of total volume 10 µl, containing 1 µl of first-strand cDNA and 0.6 µl of 10 µM of each primer. Primers used in this study are listed in Table 1. RT-qPCR efficiency and cycle threshold (CT) value were determined using the standard curves for each primer set. Efficiency-corrected transcript levels of three biological replicates for all samples were used to determine relative expression levels. The expression levels were normalized against the *poly-UBQ10* level (Cushman et al., 2008). Statistical significance was evaluated using a Student's *t*-test. Primer specificity was confirmed using a melting curve analysis after 40 amplification cycles by increasing the temperature from 60°C to 95°C. The gene number in our ice plant mRNA database for *Arabidopsis* homologous genes is as follows: *ARF19*:*Mcr014963.000*, *IAA7*: *Mcr008438.005*, *PIN2*: *Mcr005638.000*, *PIN3*: *Mcr015316.000*, *PLDP2*: *Mcr011507.001*, *PID*: *Mcr014100.002*, *LAZY3*: *Mcr015094.016*, and *LAZY5*: *Mcr002559.000*.

**Table 1.**
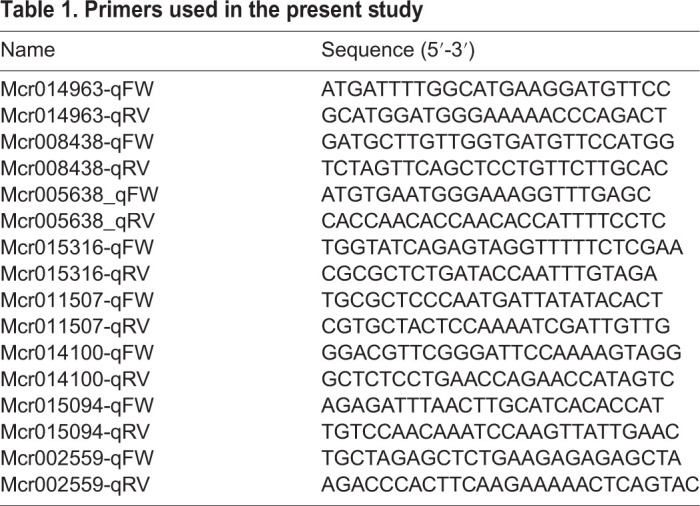
**Primers used in the present study**

## Supplementary Material

Supplementary information
