# Supplementary material for: Root system architecture analysis in Mesembryanthemum crystallinum (ice plant) seedlings reveals characteristic root halotropic response
Source: Biol Open. 2021 Mar 29;10(3):bio052142. doi: 10.1242/bio.052142 (PMC8034872; doi:10.1242/bio.052142)
Supplement: Supplementary information [file biolopen-10-052142-s1.pdf]

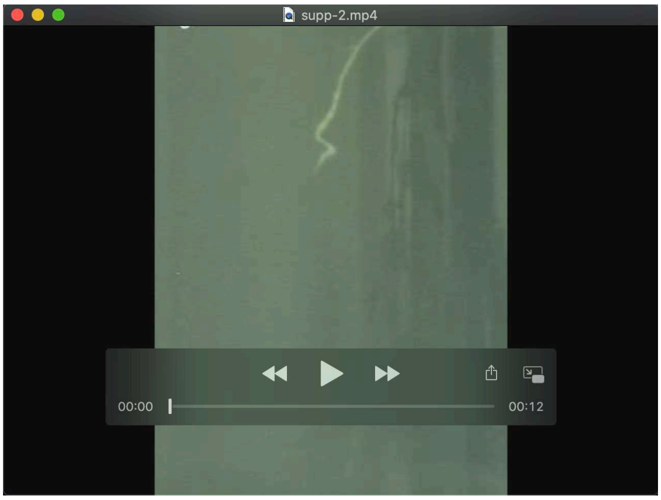

Movie 1. Time-lapse imaging of an ice plant grown in 0 mM NaCl containing medium in the glass bottle system. Movie shows an ice plant root treated with 0 mM NaCl containing Murashige-Skoog (MS) agarose medium. Images were taken every hour for 72 h. The number in the movie indicates time (h) after beginning time-lapse imaging.

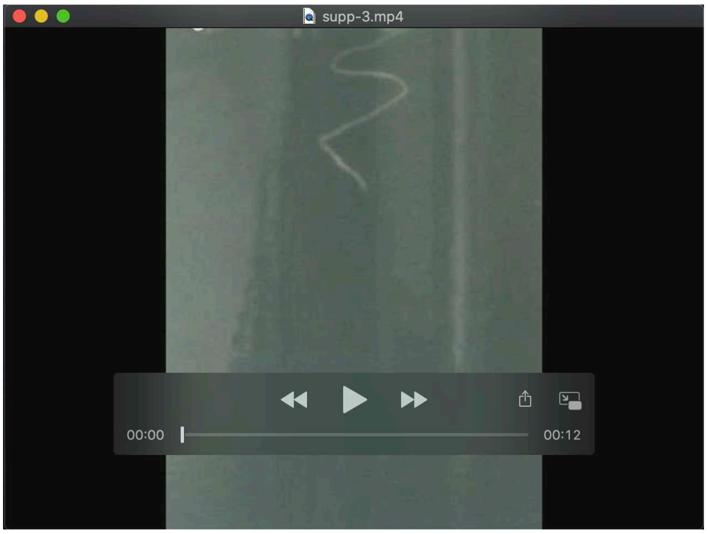

Movie 2. Time-lapse imaging of an ice plant grown in 150 mM NaCl containing medium in the glass bottle system. Movie shows an ice plant root treated with 150 mM NaCl-containing Murashige-Skoog (MS) agarose medium. Images were taken every hour for 72 h. The number in the movie indicates time (h) after beginning time-lapse imaging.

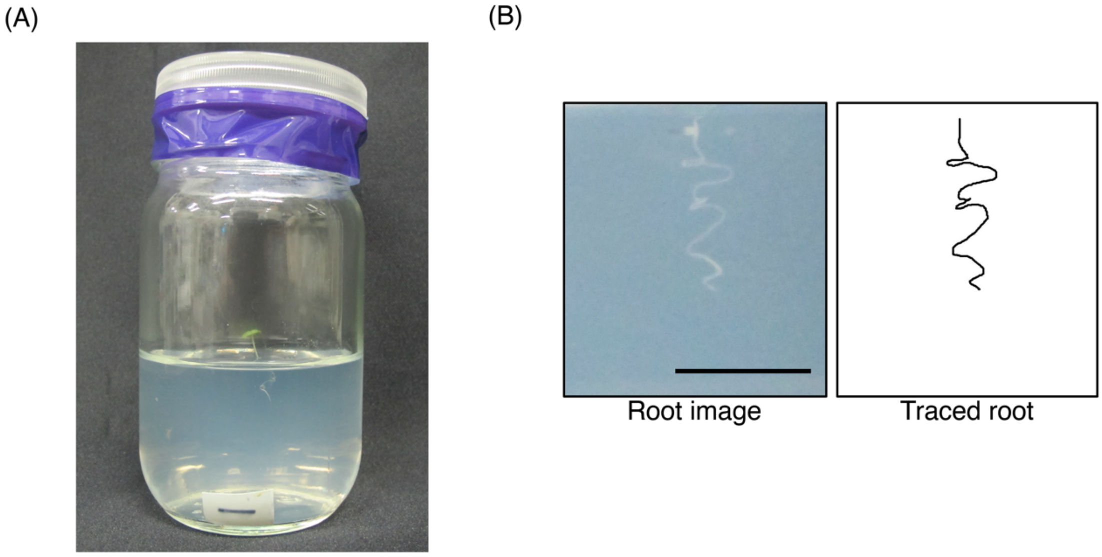

Fig. S1. Image processing for RSA analysis using GiA-roots. (A) A representative image of the glass bottle system used in this study. (B) Traced root image for GiA-roots analysis. A root picture from the glass bottle was traced using Adobe Photoshop and input in GiA-roots. Scale bar: 1 cm.

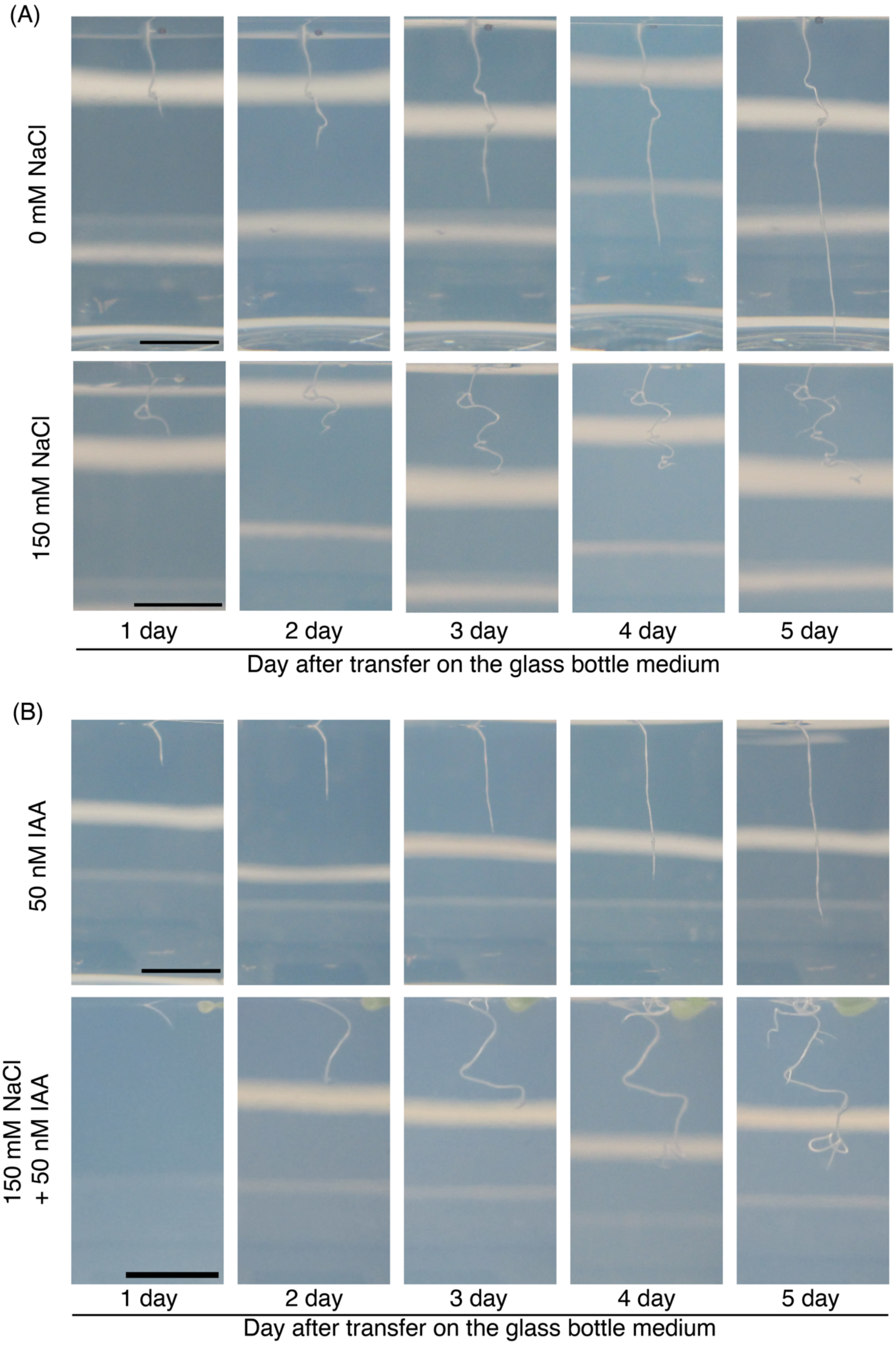
(A)
0 mM NaCl
150 mM NaCl
1 day
2 day
3 day
4 day
5 day
Day after transfer on the glass bottle medium
(B)
50 nM IAA
150 mM NaCl
+ 50 nM IAA
1 day
2 day
3 day
4 day
5 day
Day after transfer on the glass bottle medium

Fig. S2. Representative images of ice plants grown in the solid media in glass bottles. (A) Ice plant root images taken every day for 5 days after transfer of ice plant seedlings onto solid medium containing 0 mM and 150 mM NaCl in the glass bottle. (B) Ice plant root images taken every day for 5 days after transfer of ice plant seedlings onto solid medium containing 50 nM IAA and 150 mM NaCl supplemented with 50 nM IAA in the glass bottles. Scale bars: 1 cm.

Biology Open • Supplementary information
